# Supplementary material for: Getting personal with epigenetics: towards individual-specific epigenomic imputation with machine learning
Source: Nat Commun. 2023 Aug 7;14:4750. doi: 10.1038/s41467-023-40211-2 (PMC10406842; doi:10.1038/s41467-023-40211-2)
Supplement: Supplementary file 1 — Supplementary Information [file 41467_2023_40211_MOESM1_ESM.pdf]

Getting Personal with Epigenetics: Towards Machine-Learning-Assisted Precision Epigenomics

Supplementary Materials

1 Supplementary Figures

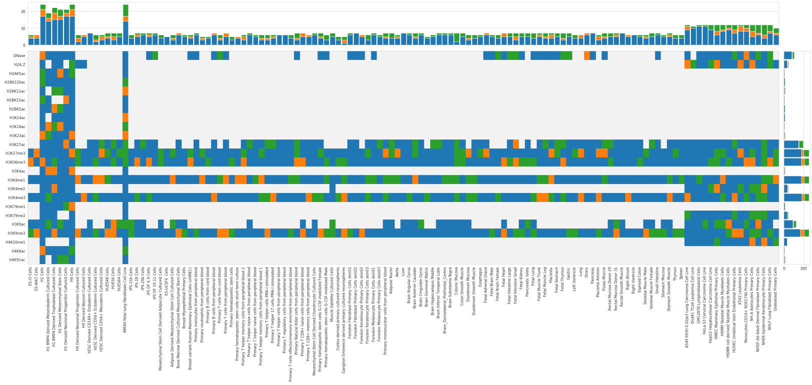

Supplementary Fig. 1 Roadmap data split. The data matrix shows which tracks from the Roadmap consortium were part of the training (blue), validation (orange) and test (green) sets.

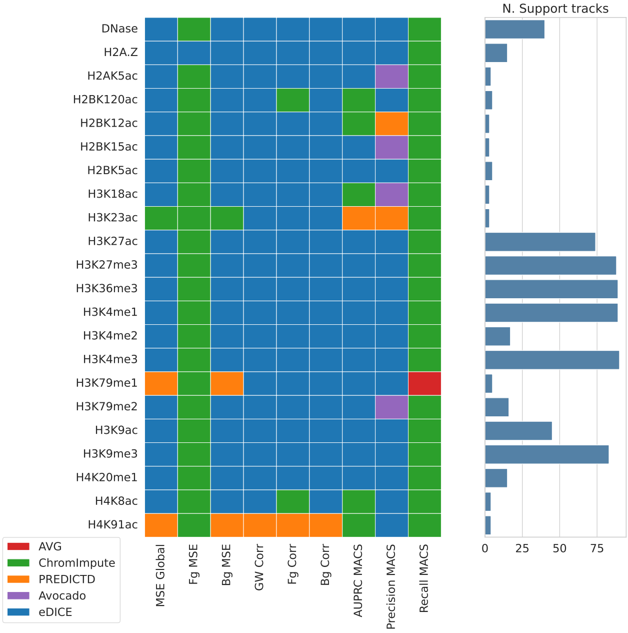

Supplementary Fig. 2 Best model for each assay. The categorical heatmap showcases which model presents the best average test performance for each assay, divided by metric.

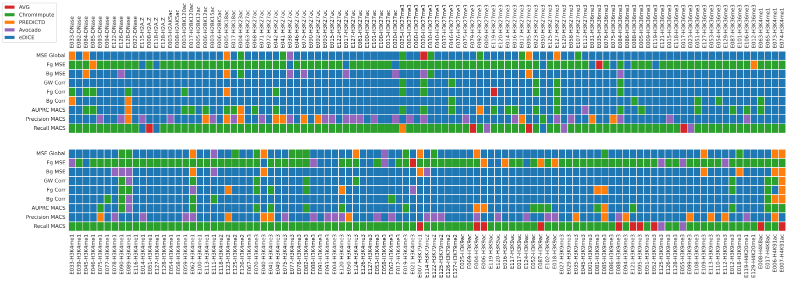

Supplementary Fig. 3 Best model for each test track. This heatmap displays the model that achieves the best performance for each assay-cell type combination in the test set, split by metric.

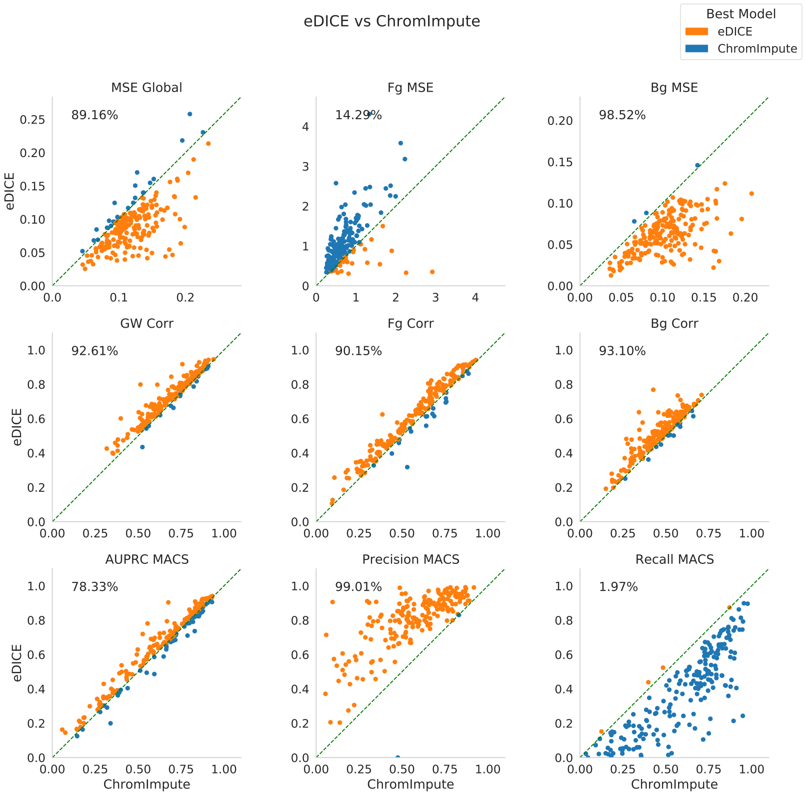

Supplementary Fig. 4 eDICE vs ChromImpute - individual test tracks. Scatter plots for the comparison of the performance of eDICE against ChromImpute for each individual test track on a subset of the evaluation metrics metric. Each point corresponds to an individual test track, whose coordinates are given by the metric of the plot for the baseline and eDICE. The percentage in the top-left corner of each plot indicates the percentage of tracks on which eDICE outperforms the baseline.

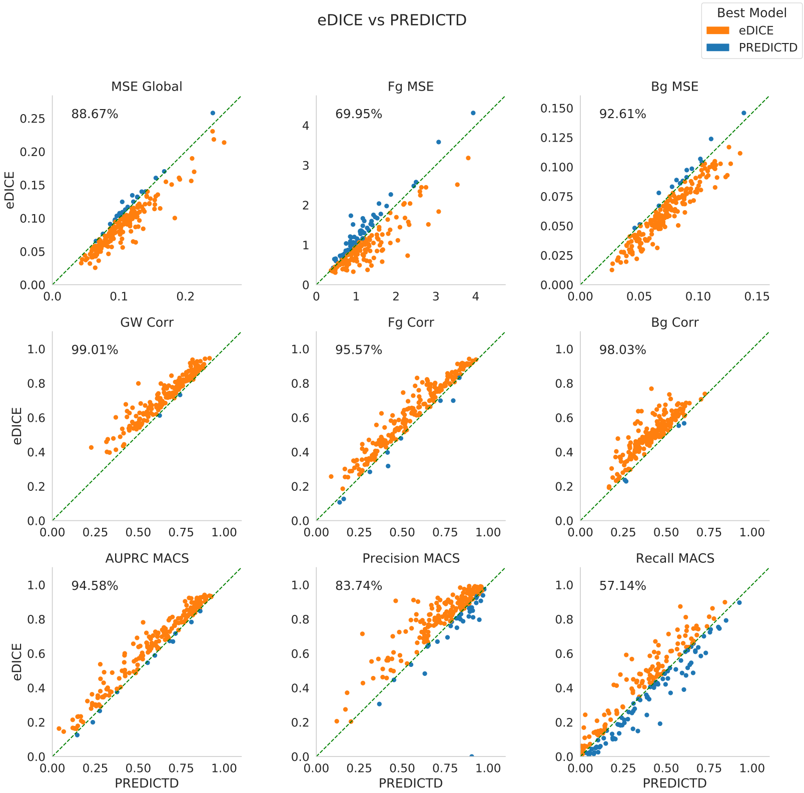

Supplementary Fig. 5 eDICE vs PREDICTD - individual test tracks. Scatter plots for the comparison of the performance of eDICE against PREDICTD for each individual test track on a subset of the evaluation metrics metric.

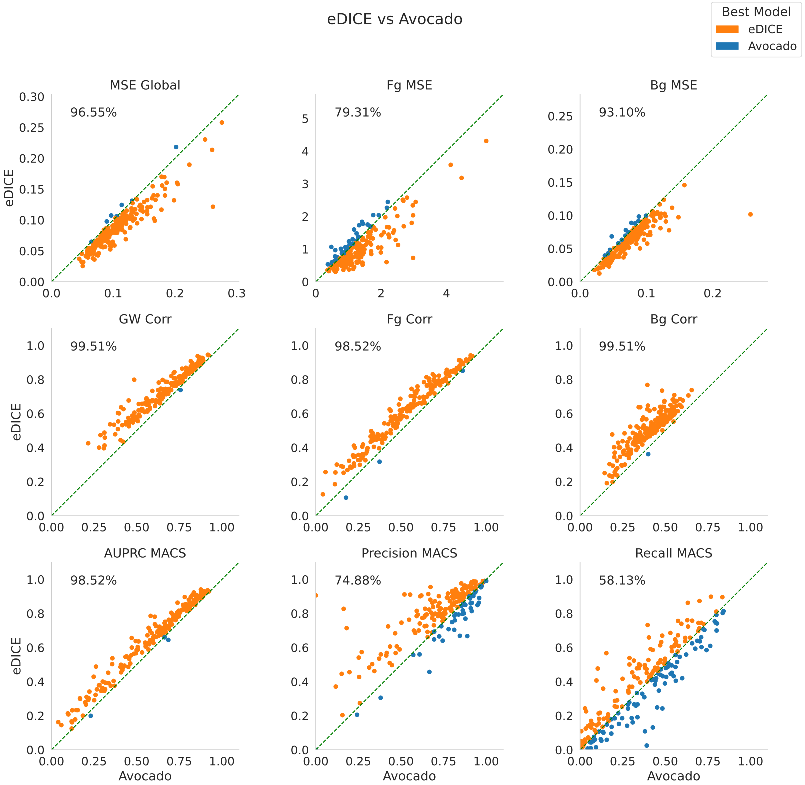

Supplementary Fig. 6 eDICE vs Avocado - individual test tracks. Scatter plots for the comparison of the performance of eDICE against Avocado for each individual test track on a subset of the evaluation metrics metric.

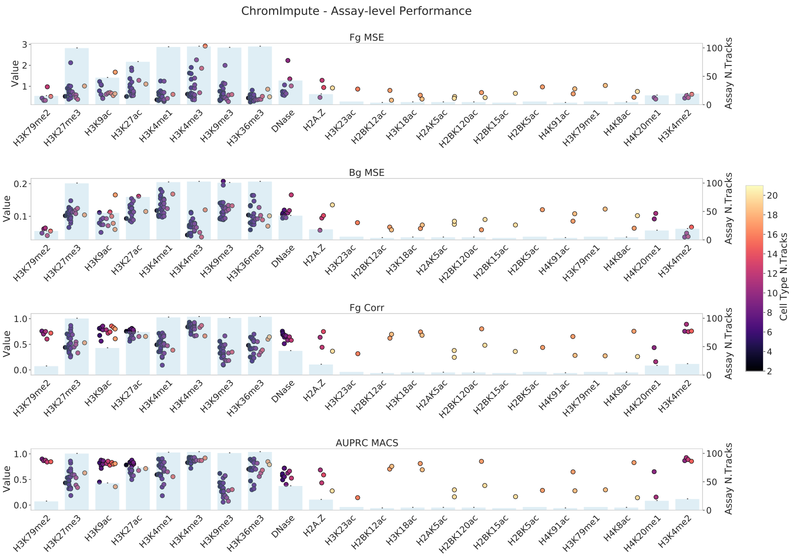

Supplementary Fig. 7 ChromImpute performance on the Roadmap test set. Track level performance of the imputations divided by assay for ChromImpute on the 203 test tracks from the Roadmap dataset.

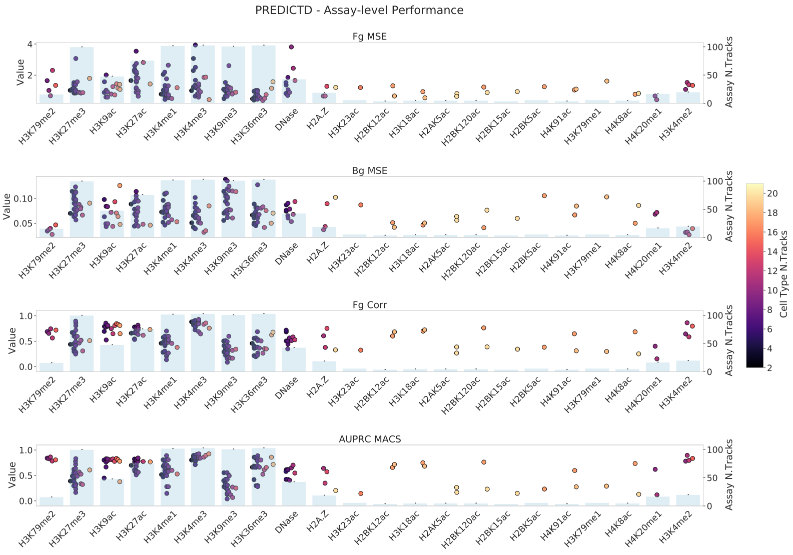

Supplementary Fig. 8 PREDICTD performance on the Roadmap test set. Track level performance of the imputations divided by assay for PREDICTD on the 203 test tracks from the Roadmap dataset.

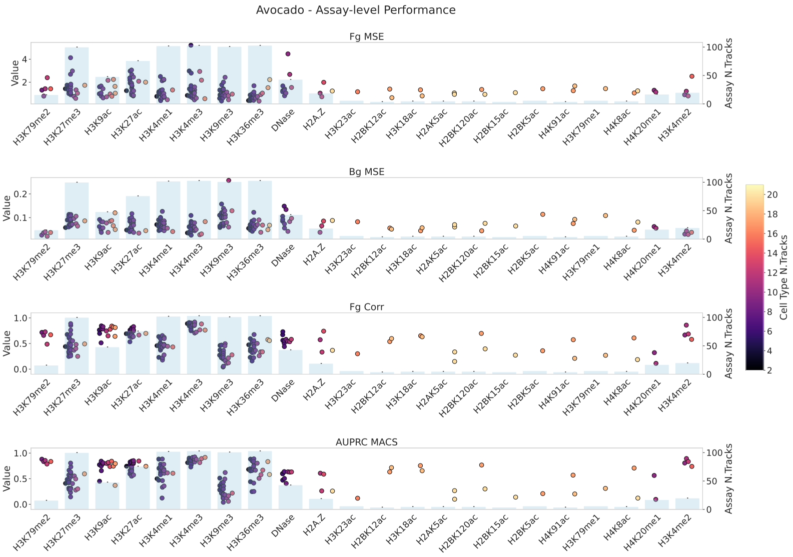

Supplementary Fig. 9 Avocado performance on the Roadmap test set. Track level performance of the imputations divided by assay for Avocado on the 203 test tracks from the Roadmap dataset.

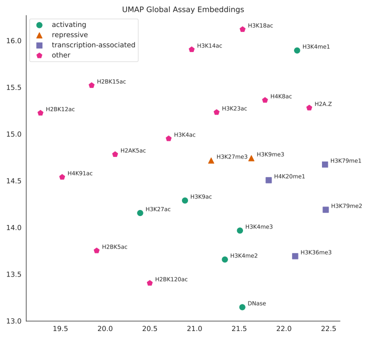

Supplementary Fig. 10 Global assay embeddings. UMAP projection of assay global embeddings

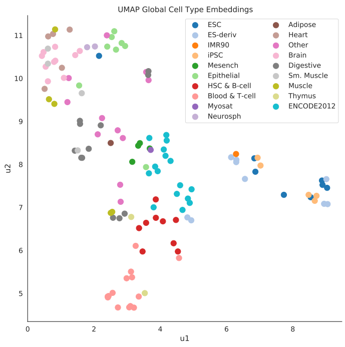

Supplementary Fig. 11 Global cell type embeddings. UMAP projection of cell type global embeddings

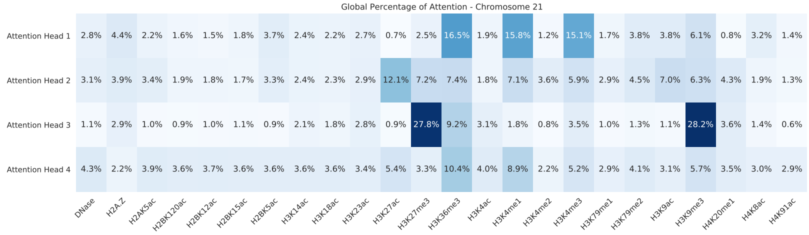

Supplementary Fig. 12 The attention heads focus on a few well mapped modifications. Percentage of attention that the 4 attention heads dedicate to each assay over the entire chromosome 21.

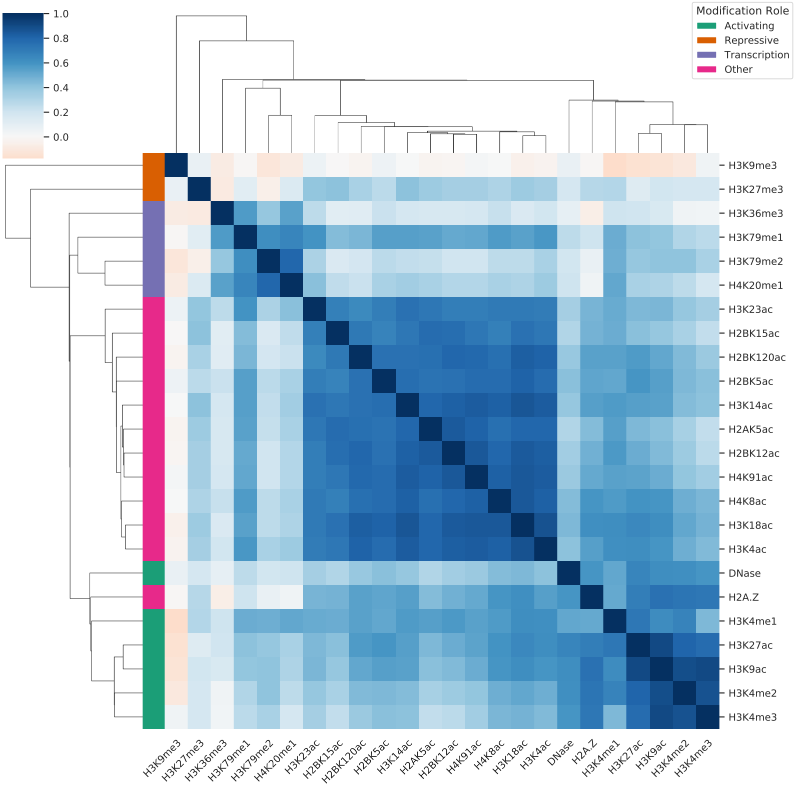

Supplementary Fig. 13 Histone modifications with shared functions show high correlation. Hierarchical clustering of the correlation matrix between epigenetic marks, calculated using the average tracks over all cell types.

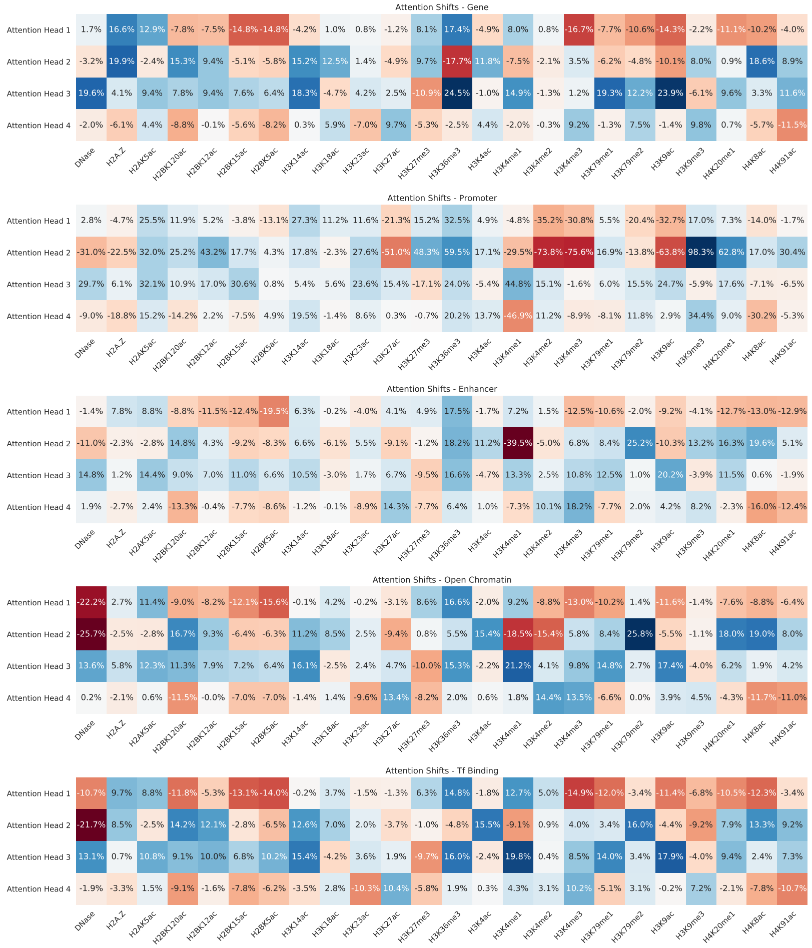

Supplementary Fig. 14 The attention weights shift with different patterns in functional regions of the genome. Differential percentage of attention that the 4 attention heads dedicate to each assay over the annotated portions of chromosome 21.

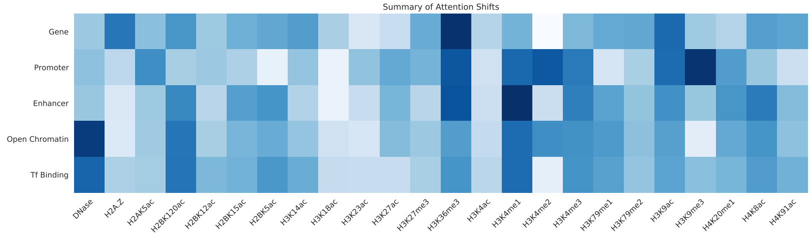

Supplementary Fig. 15 Summary of the attention shifts in the functional regions of the genome. Visual summary of the attention shifts for each assay in annotated regions of the genome.

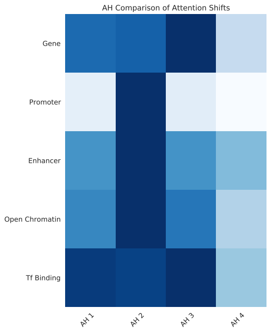

Supplementary Fig. 16 Summarized attention shifts for each transformer head. Visual summary of the attention shifts for each attention head in annotated regions of the genome.

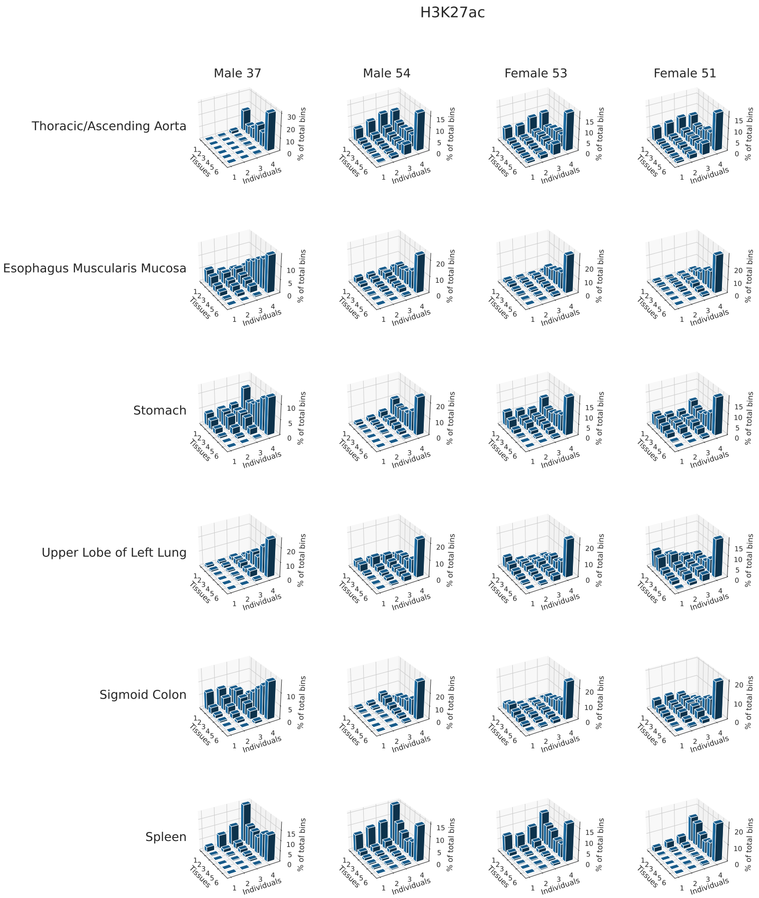

Supplementary Fig. 17 3D occupancy histogram - H3K27ac. 3D Histograms representing between how many tissues and individuals each enriched bin is shared for H3K27ac tracks in the EN-TEx dataset.

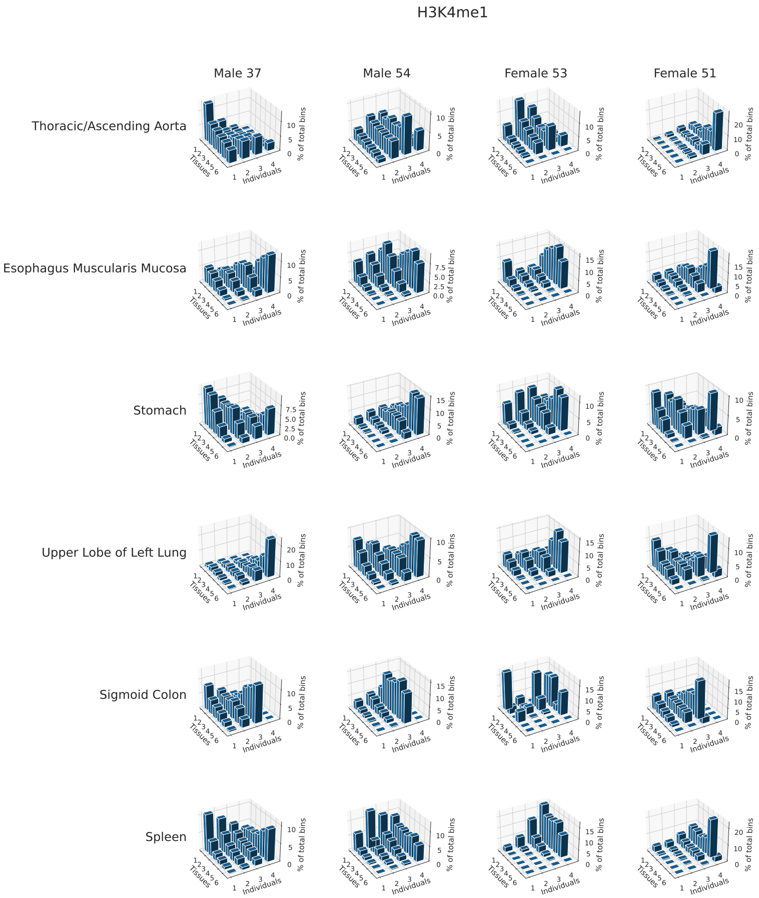

Supplementary Fig. 18 3D occupancy histogram - H3K4me1. 3D Histograms representing between how many tissues and individuals each enriched bin is shared for H3K4me1 tracks in the EN-TEx dataset.

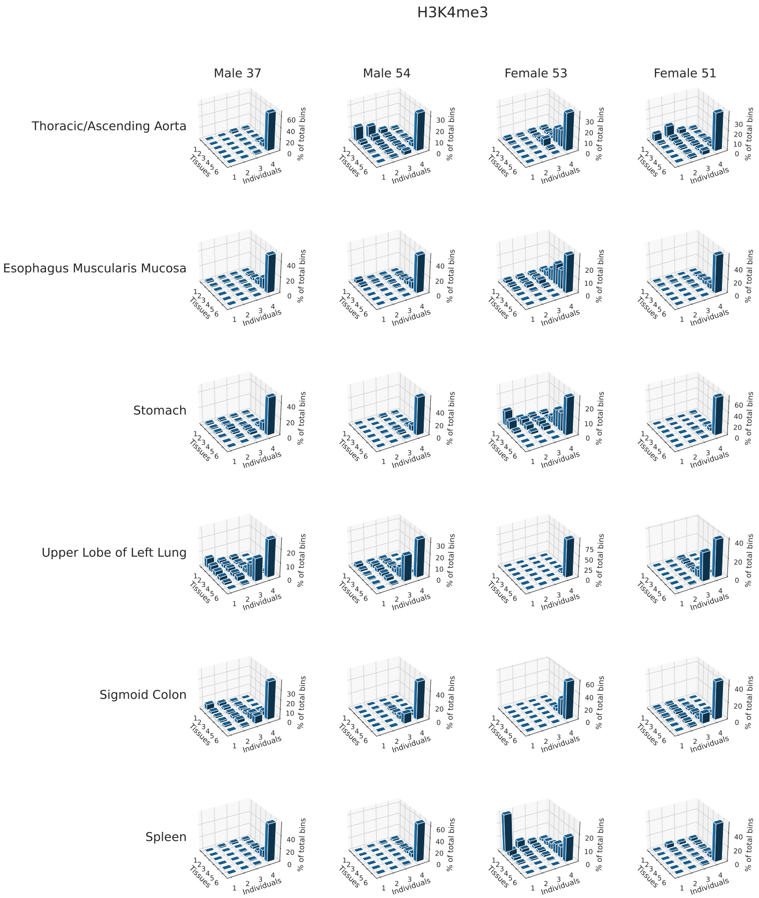

Supplementary Fig. 19 3D occupancy histogram - H3K4me3. 3D Histograms representing between how many tissues and individuals each enriched bin is shared for H3K4me3 tracks in the EN-TEx dataset.

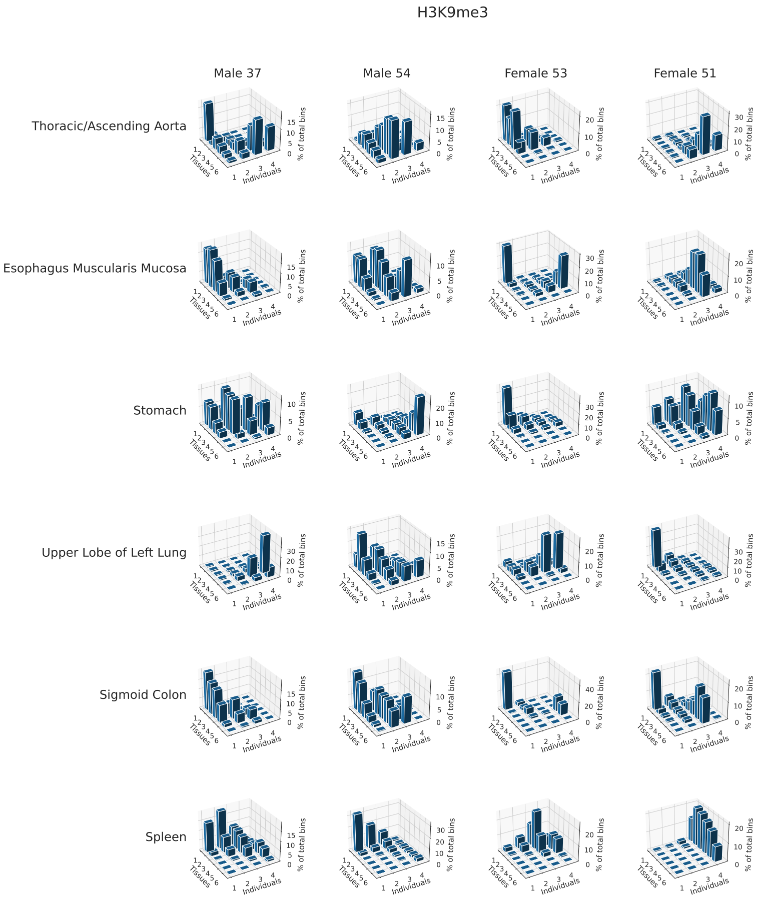

Supplementary Fig. 20 3D occupancy histogram - H3K9me3. 3D Histograms representing between how many tissues and individuals each enriched bin is shared for H3K9me3 tracks in the EN-TEx dataset.

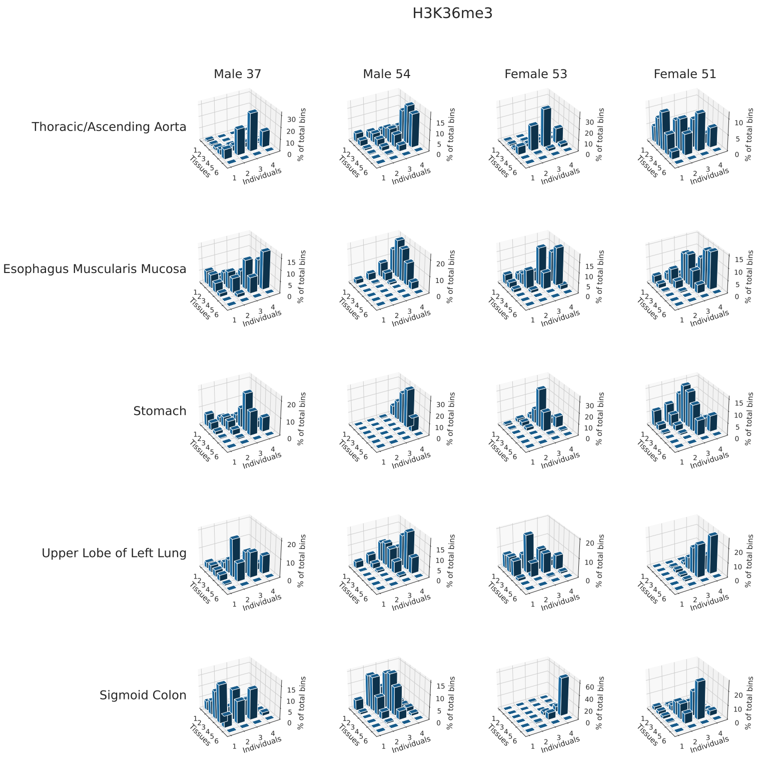

Supplementary Fig. 21 3D occupancy histogram - H3K36me3. 3D Histograms representing between how many tissues and individuals each enriched bin is shared for H3K36me3 tracks in the EN-TEx dataset.

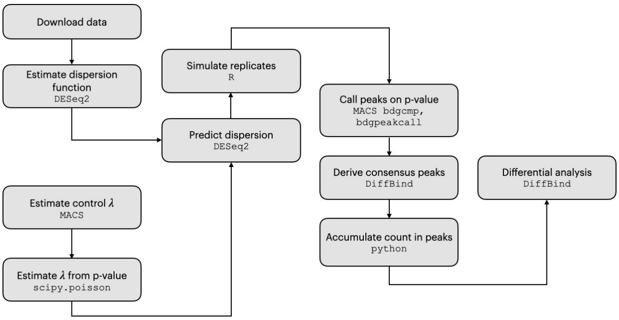

Supplementary Fig. 22 Differential analysis pipeline. Schematic of the steps performed to compare peaks between two different cell types.

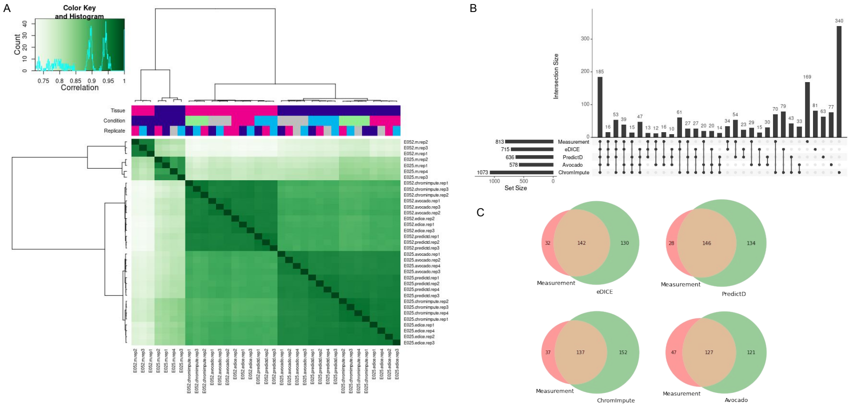

Supplementary Fig. 23 Differential peak analysis pipeline comparing different imputation methods. Only measurement samples were used to derive the consensus peakset: (a) Correlation heatmap of the affinity scores for different methods and replicates (b) Upset plot showing the size of set intersections of differentially enriched peaksets among imputation methods (c) Venn diagram showing peaks that are detected as differentially enriched between tissues using imputed and measured signals.

2 Supplementary Tables

Model	# models	# parameters per model
ChromImpute	203	/
PREDICTD	8	$\sim 11.5\,B$
Avocado	1	$\sim 3.4\,B$
eDICE	1	$\sim \mathbf{6\,M}$

Supplementary Table 1 Number of models and parameters per model required to make genome-wide predictions on the selected Roadmap test set. While PREDICTD and Avocado require several billion parameters for genome-wide prediction, eDICE requires only 6 million to obtain competitive performance.

	**Genome-Wide Reconstruction**		**MACS vs Imp. Classification**	
	GW Corr	MSE Global	AUPRC MACS	AUROC MACS
AVG	0.563 ± 0.02	0.159 ± 0.008	0.45 ± 0.029	0.931 ± 0.006
Avocado	0.668 ± 0.022	0.108 ± 0.005	0.593 ± 0.033	0.956 ± 0.005
ChromImpute	0.697 ± 0.019	0.119 ± 0.005	0.625 ± 0.032	0.967 ± 0.004
PREDICTD	0.669 ± 0.02	0.106 ± 0.005	0.592 ± 0.032	0.96 ± 0.005
eDICE	$\mathbf{0.735 \pm 0.018}$	$\mathbf{0.091 \pm 0.005}$	$\mathbf{0.651 \pm 0.031}$	$\mathbf{0.969 \pm 0.004}$

	**Background Reconstruction**		**Foreground Reconstruction**	
	Bg Corr	Bg MSE	Fg Corr	Fg MSE
AVG	0.311 ± 0.015	0.124 ± 0.005	0.467 ± 0.028	1.328 ± 0.097
Avocado	0.401 ± 0.016	0.073 ± 0.004	0.537 ± 0.03	1.283 ± 0.089
ChromImpute	0.455 ± 0.016	0.102 ± 0.004	0.571 ± 0.028	$\mathbf{0.73 \pm 0.057}$
PREDICTD	0.418 ± 0.016	0.074 ± 0.003	0.548 ± 0.029	1.189 ± 0.087
eDICE	$\mathbf{0.503 \pm 0.016}$	$\mathbf{0.063 \pm 0.003}$	$\mathbf{0.614 \pm 0.028}$	1.041 ± 0.082

	**Obs. MACS vs Imp. MACS Classification**			
	F1 MACS	MCC MACS	Precision MACS	Recall MACS
AVG	0.41 ± 0.024	0.415 ± 0.023	0.451 ± 0.027	0.451 ± 0.03
Avocado	0.45 ± 0.034	0.484 ± 0.031	0.744 ± 0.029	0.373 ± 0.033
ChromImpute	$\mathbf{0.544 \pm 0.027}$	$\mathbf{0.554 \pm 0.025}$	0.574 ± 0.029	$\mathbf{0.603 \pm 0.033}$
PREDICTD	0.453 ± 0.032	0.485 ± 0.029	0.739 ± 0.025	0.371 ± 0.031
eDICE	0.481 ± 0.034	0.52 ± 0.029	$\mathbf{0.803 \pm 0.023}$	0.392 ± 0.033

Supplementary Table 2 Performance metrics for the imputation of the 203 test tracks on chromosome 21. In bold, we highlighted the best performance for each metric. For each performance metric, we checked whether any model other than the best one failed to reject the null hypothesis of a shared mean in a two-sided paired t-test with a significance level of 0.01 (N=203). No comparable performance was found.

Tissue	Assay	ENCDO845WKR (male 37 years old)	ENCDO451RUA (male 54 years old)	ENCDO793LXB (female 53 years old)	ENCDO271OUW (female 51 years old)
ascending aorta	H3K27ac			ENCFF472VCY	ENCFF002VCM
	H3K36me3			ENCFF529EEQ	ENCFF602IDJ
	H3K4me1			ENCFF971TZV	ENCFF843GKY
	H3K4me3			ENCFF229NZH	ENCFF383JQQ
	H3K9me3			ENCFF616UAG	ENCFF644UVB
thoracic aorta	H3K27ac	ENCFF652JBU	ENCFF344YDV		
	H3K36me3	ENCFF700ZLW	ENCFF556REM		
	H3K4me1	ENCFF141KEC	ENCFF388BWY		
	H3K4me3	ENCFF327VEH	ENCFF723SJI		
	H3K9me3	ENCFF221YER	ENCFF198OYC		
esophagus muscularis mucosa	H3K27ac	ENCFF969PNR	ENCFF437XOA	ENCFF282WYM	ENCFF707HDC
	H3K36me3	ENCFF476ILL	ENCFF567TNH	ENCFF723KNC	ENCFF861YVB
	H3K4me1	ENCFF295FVH	ENCFF478GEN	ENCFF067DEH	ENCFF877IZN
	H3K4me3	ENCFF280VIW	ENCFF979ERR	ENCFF156BAJ	ENCFF636QVZ
	H3K9me3	ENCFF501FLV	ENCFF380LRK	ENCFF885KSL	ENCFF729RCC
stomach	H3K27ac	ENCFF440VXQ	ENCFF241NFR	ENCFF134NHD	ENCFF140JKH
	H3K36me3	ENCFF302OSD	ENCFF414FQR	ENCFF428LSW	ENCFF441GAP
	H3K4me1	ENCFF140RPG	ENCFF034VHX	ENCFF411ZSD	ENCFF131UFO
	H3K4me3	ENCFF175WCH	ENCFF221JKN	ENCFF309DCR	ENCFF468UJW
	H3K9me3	ENCFF285GPE	ENCFF765FRJ	ENCFF674DMF	ENCFF554RBC
upper lobe of left lung	H3K27ac	ENCFF204PWD	ENCFF305XCG	ENCFF071QUR	ENCFF178DCM
	H3K36me3	ENCFF549FDA	ENCFF572VAG	ENCFF267DPL	ENCFF700BQU
	H3K4me1	ENCFF076BNQ	ENCFF614CUN	ENCFF777AOP	ENCFF344GZI
	H3K4me3	ENCFF898DYH	ENCFF526RLU	ENCFF887IUV	ENCFF578OBF
	H3K9me3	ENCFF672QOH	ENCFF583KBZ	ENCFF076RRH	ENCFF317KCA
sigmoid colon	H3K27ac	ENCFF669TSI	ENCFF507VUO	ENCFF156QWL	ENCFF497GAF
	H3K36me3	ENCFF284VTI	ENCFF312AHC	ENCFF577WWK	ENCFF744WIO
	H3K4me1	ENCFF686SIG	ENCFF427WMO	ENCFF126IMK	ENCFF113YOI
	H3K4me3	ENCFF892GNP	ENCFF445QHF	ENCFF905JEN	ENCFF135XHH
	H3K9me3	ENCFF811EKT	ENCFF925APW	ENCFF782WTP	ENCFF477NHW
spleen	H3K27ac	ENCFF230ZQJ	ENCFF786NLG	ENCFF735GCH	ENCFF702AJP
	H3K4me1	ENCFF873LAX	ENCFF049HBP	ENCFF402YZU	ENCFF599IBO
	H3K4me3	ENCFF422ZXF	ENCFF189KXE	ENCFF656AAS	ENCFF462VQE
	H3K9me3	ENCFF952NOQ	ENCFF418KYC	ENCFF447CEL	ENCFF088KFT

Supplementary Table 3 EN-TEx accession codes. Accession codes for the 116 selected tracks from the EN-TEx dataset used in the imputation of personalized epigenomes. The measurements for "thoracic aorta" and "ascending aorta" were merged into a single tissue label "aorta".

3 Supplementary Notes

3.1 Evaluation Strategy

Measuring the performance of an imputation model is far from trivial. Properly designing an evaluation strategy is crucial to ensure that the results reflect the workings of the models and that the comparison to a selected set of baselines aligns with the use case intended for the model.

Ernst and Kellis [16] validated the performance of ChromImpute with a leave-one-out (LOO) strategy, where all tracks short of the one specific target test track are used during different training instances. This strategy provides a substantial prediction advantage due to the optimal number of training data sets. However, it may not give the best assessment of the method's generalization ability [2]. In contrast, the authors of PREDICTD and Avocado have analysed the imputed values for a hold-out set of test tracks using 5-fold cross-validation. This procedure has also been adopted in a recent ENCODE Imputation Challenge [47], and we will do the same in this work. The split of the data is identical to one of the folds used in [19]. Supplementary Figure 1 depicts a data matrix showing which tracks from the Roadmap consortium were part of the training, validation and test sets. Quality control of the Roadmap Consortium guarantees a minimum standard for all data sets [15].

In previous work, including the Encode Imputation Challenge, training, validation and test tracks are processed following established computational

pipelines[1]. This pipeline includes a pooling step for biological and technical replicates and subsampling to maximum library size. Notably, the MACS2 peak caller is applied to calculate the statistical significance of enrichment at each base pair in the genome. The resulting genome-wide signal tracks containing the statistical significance of enrichment (i.e., the $-log10$ p-values) at each base pair in the genome have been used both as input training data and to validate imputations [19]. Recently, however, it has been demonstrated that MACS2 outputs biased P-values and false discovery rate estimates that can be many orders of magnitude too optimistic [5]. This systematic bias raises several problems; for instance, there is no sound basis for choosing a p-value cut off for reporting results. Additionally, as the introduced bias depends on the ChIP-Seq data from which the p values are computed, there can be "distributional shifts" between training and test sets, as observed in the Encode Imputation Challenge.

To reduce the impact of possible systematic biases, we employ a varied set of performance metrics, each aiming to capture different facets of the imputation task. For example, comparing models using Area-Under-Curve metrics ensures that choosing a specific p-value cut-off is not the determining factor in ranking the imputation models.

3.2 Performance Metrics

An appropriate choice of metrics is essential for robust comparisons between imputation models. The most immediate and intuitive approach from a machine learning point of view is to measure the ability of a model to reconstruct genome-wide signals (i.e. corrected -log10 p' values). Commonly used measures include genome-wide mean-squared-error (GW MSE) and genome-wide Pearson correlation (GW Corr) [19]. Importantly, however, most Histone modifications are only significantly enriched on a small subset of genomic bins. The remaining bins have low background signals, which are strongly determined by experimental conditions, including unspecific binding of the antibody. As these background regions are over-represented on the genome, they easily dominate genome-wide assessment measures.

Durham et al. [18] therefore defined additional performance measures, including MSE1obs, which measures the MSE on the top 1% of the positions according to the ChIP-seq signal; MSE1imp measures the MSE on the top 1% of the positions according to the imputed signal. On the other hand, outside the imputation literature, it is a common strategy to analyse histone modification measurements by focusing on foreground regions which are identified using Peak callers such as MACS2 [22]. In this case, significant enriched regions are merged if they are close together and fluctuations below a given threshold are tolerated within a foreground peak if they are small enough. Additionally, regions of significant enrichment have to have a minimum length (at least as big as the fragment length) to be called foreground peaks. Peak callers therefore provide a more robust segmentation of the genome into foreground

[1] e.g. https://github.com/ENCODE-DCC/chip-seq-pipeline2.

and background regions. Here, we follow a hierarchical analysis to evaluate the imputations: First, we test the ability of the model to correctly classify genomic regions into foreground and background regions (e.g. [8]). The signal reconstruction potential of the models is then separately analysed for bins categorized as foreground and background. For the foreground regions, we are additionally interested in how well the peak intensities are predicted (e.g. [9]), and whether the shape of the signal in the peak is well captured by the prediction. (e.g.[10, 24]).

3.3 Validation on the Roadmap Reference Epigenome

Table 2 presents the numerical results for the imputation of the test tracks of the Roadmap dataset. For each metric, we highlighted the best-performing model, as well as the competitors for which a 2-sided t-test failed to reject the null hypothesis with a significance threshold of 0.01 (N=203).

Comparing eDICE to the baselines at the assay level (Figure 2) and at the individual track level (Figures 3, 4, and 5) highlights that the reported improvements are consistent across the board, with the notable exception of four metrics in which ChromImpute outperforms eDICE (Fg MSE, Precision MACS, F1 MACS, and MCC MACS). For all four of them, the underlying issue is that factorization models such as eDICE and PREDICT consistently underestimate the height of peaks, leading to reduced detection of enriched regions. As a consequence, eDICE is more conservative than ChromImpute in its prediction, a fact that is mirrored in the Recall MACS metrics.

Different assays present different overall imputation performance. For example, in the evaluation of eDICE, H3K9me3 and H3K27me3 displayed consistently suboptimal performance. Figures 7 and 8 highlight how this tendency is not exclusive to eDICE, but shared by the baseline models.

3.4 Differential peak analysis

Differential peak analysis aims to detect meaningful differences between biological samples and typically involves multiple replicates in each group of interest. The inclusion of multiple samples enables the discrimination of natural variability within cell types from differentially enriched regions caused by the biological differences between tissues.

To analyse the use of imputed signals for detecting differential enrichment, we chose the H3K9ac assays for cell types E025 (Adipose Derived Mesenchymal Stem Cell Cultured Cells) and E052 (Muscle Satellite Cultured Cells) as samples for a case study. These two epigenomic tracks are part of the hold-out test set, share a comparable number of tracks for the two tissues in the training, validation, and test sets, and have 4 and 3 measured replicates, respectively.

We tested the use of imputations for differential analysis with the DiffBind package [25]. DiffBind requires specifying the peak set and providing the aligned reads for treatment and control in a sample sheet. For the measurement group, we use the data from the Roadmap consortium, which includes

the aligned reads for each replicate in *.tagAlign* format and the peaks called with MACS2 in *.narrowPeak* format. In addition, each replicate is associated with a cell-type-specific control track. The aligned *.tagAlign* files are converted to BAM format with BedTools [13].

For the imputed tracks, we devised a procedure to simulate replicates from the predicted p-value signal. Such a procedure is an approximation that requires several assumptions, yet it suffices to provide a proof of concept for using imputation models in bioinformatics software pipelines. Nevertheless, for such applications, future imputation models would likely need to focus on modelling not only the average value of a signal but also the uncertainty involved.

To simulate replicates using p-value signals, we assume that the read counts at each genomic position follow a negative binomial distribution, a common model used for sequencing data [14–16]. To draw samples from this distribution, we need to estimate two parameters, mean and dispersion.

We estimate the mean parameter by inverting the procedure used to generate the p-value tracks. The p-value signal results from the Survival Function of a background Poisson model. The genomic-position specific mean parameter of the background distribution is calculated from tissue-specific control samples available in the Roadmap dataset. Using the parameters of the background distribution, the read counts for a given track are obtained through the Inverse Survival Function (ISF).

To infer the dispersion parameters for each cell type, we made use of the software package DESeq2 [15]. Specifically, DESeq2 is capable of fitting a function that, given the mean value as input, can output the dispersion value. We selected tracks from the training set for H3K9ac whose tissues present sufficient similarity to the target tissues (E023 for E025 and E107-E108 for E052). These selected tracks are used to estimate the mean-dispersion functions, which are then used on the previously estimated mean parameters to generate bin-wise dispersion parameters.

Given these bin-specific parameters derived from the imputed signals, we generate three samples per cell type by sampling from the negative binomial distribution.

The MACS[22] command *bdgcmp* and *bdgpeakcall* were used to generate the peak set on the p-value track for each cell type in both measurement and simulated replicates. Next, the consensus peakset for all samples was estimated using DiffBind. ENCODE blacklisted regions[17] were removed from the consensus peakset. Aggregated counts for these consensus peaks were computed for both measurement and simulated replicates, and were directly read into the DiffBind DBA object with *dba.peakset* function. Using a False Discovery Rate (FDR) threshold of 0.05, we determined the differentially enriched peaks for both measurements and imputations. An overview of the differential peak analysis pipeline can be found in 23.

4 Interpreting the model

4.1 Global Embeddings

eDICE learns global embeddings that capture the high-level relationships between assays and cell types, which we can display through a UMAP [18] projection.

Epigenetic modifications that are generally associated with the same functionality tend to cluster together in the learned representations (Figure 10). Assigning global roles to epigenetic modifications neglects much of the nuance of the regulatory mechanisms of the genome. Nonetheless, we can consider some general patterns.

H3K9ac, H3K4me2, H3K4me3 are modifications typically associated with active promoters [19, 20], while H3K4me1 and H3K27ac (and partially H3K4me2) are linked with active enhancers [20, 21]. We classify these marks, together with the DNAse-seq assay [22], under the broad label of *activating* modifications.

H3K27me3 is an antagonist to H3K27ac and is broadly associated to gene repression together with H3K9me3 [23, 24].

H3K36me3, H4K20me1, H3K79me1 are linked to exons [25], and we consider these modifications under the label of *transcription-associated* together with H3K79me2, which is associated with active gene bodies and alternative splicing [26, 27].

The partitioning of the epigenetic marks reflects the correlation patterns that emerge from the observed data. We calculated the average tracks for each assay across all cell types and performed agglomerative hierarchical clustering on these tracks. The results, summarised in Supplementary Figure 13, show that modifications with shared global functions tend to cluster together.

Likewise, eDICE is capable of learning the broad similarities between tissue types through the global cell embeddings represented in Figure 11.

4.2 Measures of Attention

Inspired by the analysis of attention mechanisms in protein language models [28], we define the portion of attention that each attention head h dedicates to a specific assay a in the set of observed assays A over a portion g of the genome as:

$$p_h(\alpha) = \sum_{g \in G} \sum_{a \in A} w_{a\alpha}^{(g,h)} / \sum_{g \in G} \sum_{a \in A} \sum_{a' \in A} w_{aa'}^{(g,h)} \tag{1}$$

where $w_{a\alpha}^{(g,h)}$ denotes the attention weight for the key assay α given the query assay a in the genomic bin g for attention head h. Within the formalism of key-value-query attention in Transformer models, this definition of portion of attention corresponds to the average attention given to each key. Due to the softmax function used in the calculation of the attention weights in the scaled dot-product attention, the portion of attention adds up to one over the

space of all assays for a single attention head, which makes it suitable to be expressed as a percentage of the total attention of the attention head.

To evaluate how the state of the attention blocks changes in correspondence with the functional regions of the genome, we calculated of the attention percentage while restricting the genomic bins to the aforementioned annotated regions and evaluated the relative difference in the percentage of attention dedicated to the assays compared to the global pattern in the chromosome. We define this *attention shift* for a given region R of the genome as:

$$d_h^{(R)}(\alpha) = \frac{p_h(\alpha)|_{G \equiv R} - p_h(\alpha)}{p_h(\alpha)} \tag{2}$$

where R is any type of annotated region of the genome, e.g. promoters or enhancers.

4.3 Interpreting the Attention Weights

Attention models assign a weight to the relationship between elements in an input set or sequence, which is often considered a proxy to interpret the underlying interactions between said elements. This area of research is mainly active within the application of Transformer models to Natural Language Processing (NLP) tasks [29, 30], with a strong focus on the visualization of the attention weights (e.g. [31]).

Despite this excitement, recent works have begun to highlight the limitations of attention as an explanation method, showing that in specific contexts, attention weights are at best noisy predictors of the importance of input components for a model [32] or outright misleading and weak to adversarial attacks [33]. Although these studies present some critical limitations, such as the focus on NLP models or the comparison to alternative measures of feature importance which are not necessarily valid as "ground truth", we believe caution in the analysis of attention weights to be warranted. Adding these considerations to the non-deterministic result of training attention models, we consider the analysis presented here as a diagnostic exploration rather than a detailed claim on the biology examined by the model.

The results presented are derived from the same instance of eDICE used for the reference epigenome validation. Although the details vary due to the stochastic training of the attention block, we observed that the conclusions drawn tend to hold across training instances.

We specifically focus on the attention block used for the contextualization of the assay embeddings. The attention measures employed (detailed in Supplementary Section 4.2) are based on averages of attention weights, which paint a higher-level picture than comparisons of individual coefficients.

To obtain a general overview of the eDICE model, we calculated the percentage of attention given by each head to each assay for the entire chromosome 21. These results, displayed in Figure 12, highlight how at least two of the attention heads consistently dedicate a significant portion of attention

to just a few histone modifications. These marks are among the core modifications extensively mapped across many cell types in the Roadmap dataset, i.e. H3K27ac, H3K36me3 H3K9me3, H3K4me1, and H3K4me3.

Interestingly, this phenomenon proved quite consistent over different experiments, with minor variations such as focusing on H3K27me3 rather than H3K27ac. Overall, this result suggests that the model relies extensively on marks for which a large amount of information is present during training.

To further explore the workings of the attention layer in eDICE, we gathered genomic annotations for chromosome 21 from the Ensembl [34] version 104 GRCh37 assembly for protein-coding gene bodies, enhancers, promoters, open chromatin, and transcription factors binding sites. We calculated how the percentage of attention shifts within these functional regions of the genome and displayed the results in Figure 14.

Overall, these attention shifts reveal known patterns from the literature yet present unexplained peculiarities, such as the significant shift within enhancer regions for H3K4me1, a known chromatin hallmark for these functional regions. Intuitively, one would expect the attention weight for H3K4me1 to grow within enhancers, given its biological importance, yet we observe the opposite, especially for attention head 2. Nevertheless, this pattern is inconsistent for such a setting: H3K36me3, a histone modification enriched on the gene body region associated with active gene transcription [35], shows a positive average attention shift within protein-coding genes.

Much remains to be understood about the workings of attention models. Therefore, we limit our analysis to the qualitative consideration that the larger attention shifts within functional regions correspond to known epigenetic marks that characterize said regions. Examples of these patterns include the DNase shifts for open chromatin regions [36], the shifts in DNase and H3K4me1 for transcription factor binding sites [37], and the shifts within promoters for H3K4 methylation [38], H3K9ac, H3K27ac, and their antagonists H3K9me3 and H3K27me3 [19].

We include two high-level visual summaries of the attention shifts in Figures 15 and 16, in which the absolute value of the attention shifts was summed up over the attention heads and the assays, respectively, and then 0-1 normalized for each annotated category. The resulting heatmaps provide at a glance an overview of which assays present the most considerable relative changes and which attention heads are significantly affected within functional regions of the genome.

Interestingly, it appears that the attention heads specialize at least partially, with attention head 2 being the most affected in promoters, enhancers, and open chromatin regions. In contrast, attention head 3 presents the most significant shifts for protein-coding gene bodies and transcription factor binding sites. The scarce shifts for attention head 4 also indicate that this head is not contributing significantly to the final prediction, a known phenomenon in the use of multi-headed attention models, and could be a candidate for pruning [39].

5 ENCODE imputation challenge model

An early version of our model was used as the basis of our third-placed entry in the 2019 ENCODE Imputation Challenge [40]. This model follows Avocado in adopting the factorized structure of Equation 1, but instead of directly parametrizing genomic bin representations $\mathbf{b}_k$, computes non-linear, low dimensional transformations of the input signal at each bin,

$$\hat{Y}_{ijk} = g_\theta(\mathbf{c}_i, \mathbf{a}_j, f_\phi(\mathrm{vec}(Y^k))) \quad , \tag{3}$$

where $\mathrm{vec}(Y^k)$ is a fixed-size vector whose entries are the signal values for all observed tracks at the *kth* bin, together with zeros for all unobserved tracks. In practice, the functions g_θ and f_ϕ are both implemented with two-layer MLPs, and $\mathbf{c}_i$ and $\mathbf{a}_i$ are learned cell and assay embeddings, equivalent to the 'global embeddings' $\mathbf{u}_{c_i}$ and $\mathbf{u}_{a_i}$ described in the Online Methods (Equations 4 and 6). This model can be seen as a kind of denoising autoencoder in which the decoder has a factorized structure, inherited from Avocado, allowing it to impute signal values for previously unobserved cell-assay combinations. In contrast, our model is fully factorized, replacing a single global transformation of the input signal with cell- and assay-dependent transformations of the input signal, learned via self-attention (Equation 2).

The challenge model is used as a strong baseline in our experiments, since it has demonstrated competitive performance with state-of-the-art methods including Avocado in the blind-test setting of the ENCODE Imputation Challenge.

Supplementary References

[1] Ernst, J., Kellis, M.: Large-scale epigenome imputation improves data quality and disease variant enrichment. Nature biotechnology **33**(4), 364 (2015)

[2] Schreiber, J., Bilmes, J., Noble, W.S.: Prioritizing transcriptomic and epigenomic experiments using an optimization strategy that leverages imputed data. Bioinformatics **37**(4), 439–447 (2021)

[3] Schreiber, J., Durham, T., Bilmes, J., Noble, W.S.: Avocado: a multi-scale deep tensor factorization method learns a latent representation of the human epigenome. Genome biology **21**(1), 1–18 (2020)

[4] Kundaje, A., Meuleman, W., Ernst, J., Bilenky, M., Yen, A., Heravi-Moussavi, A., Kheradpour, P., Zhang, Z., Wang, J., Ziller, M.J., *et al.*: Integrative analysis of 111 reference human epigenomes. Nature **518**(7539), 317–330 (2015)

[5] Chitpin, J.G., Awdeh, A., Perkins, T.J.: Recap reveals the true statistical significance of chip-seq peak calls. Bioinformatics **35**(19), 3592–3598 (2019)

[6] Durham, T.J., Libbrecht, M.W., Howbert, J.J., Bilmes, J., Noble, W.S.: Predictd parallel epigenomics data imputation with cloud-based tensor decomposition. Nature communications **9**(1), 1–15 (2018)

[7] Zhang, Y., Liu, T., Meyer, C.A., Eeckhoute, J., Johnson, D.S., Bernstein, B.E., Nusbaum, C., Myers, R.M., Brown, M., Li, W., *et al.*: Model-based analysis of chip-seq (macs). Genome biology **9**(9), 1–9 (2008)

[8] Tilgner, H., Nikolaou, C., Althammer, S., Sammeth, M., Beato, M., Valcárcel, J., Guigó, R.: Nucleosome positioning as a determinant of exon recognition. Nature structural & molecular biology **16**(9), 996 (2009)

[9] Karlić, R., Chung, H.-R., Lasserre, J., Vlahoviček, K., Vingron, M.: Histone modification levels are predictive for gene expression. Proceedings of the National Academy of Sciences **107**(7), 2926–2931 (2010)

[10] Kundaje, A., Kyriazopoulou-Panagiotopoulou, S., Libbrecht, M., Smith, C.L., Raha, D., Winters, E.E., Johnson, S.M., Snyder, M., Batzoglou, S., Sidow, A.: Ubiquitous heterogeneity and asymmetry of the chromatin environment at regulatory elements. Genome research **22**(9), 1735–1747 (2012)

[11] Schweikert, G., Cseke, B., Clouaire, T., Bird, A., Sanguinetti, G.: Mmdiff: quantitative testing for shape changes in chip-seq data sets. BMC

genomics **14**(1), 1–17 (2013)

[12] Stark, R., Brown, G., et al.: Diffbind: differential binding analysis of chip-seq peak data. R package version **100**(4.3) (2011)

[13] Quinlan, A.R., Hall, I.M.: Bedtools: a flexible suite of utilities for comparing genomic features. Bioinformatics **26**(6), 841–842 (2010)

[14] Robinson, M.D., Smyth, G.K.: Moderated statistical tests for assessing differences in tag abundance. Bioinformatics **23**(21), 2881–2887 (2007)

[15] Love, M.I., Huber, W., Anders, S.: Moderated estimation of fold change and dispersion for rna-seq data with deseq2. Genome biology **15**(12), 1–21 (2014)

[16] Robinson, M.D., McCarthy, D.J., Smyth, G.K.: edger: a bioconductor package for differential expression analysis of digital gene expression data. Bioinformatics **26**(1), 139–140 (2010)

[17] Amemiya, H.M., Kundaje, A., Boyle, A.P.: The encode blacklist: identification of problematic regions of the genome. Scientific reports **9**(1), 1–5 (2019)

[18] McInnes, L., Healy, J., Melville, J.: Umap: Uniform manifold approximation and projection for dimension reduction. arXiv preprint arXiv:1802.03426 (2018)

[19] Karmodiya, K., Krebs, A.R., Oulad-Abdelghani, M., Kimura, H., Tora, L.: H3k9 and h3k14 acetylation co-occur at many gene regulatory elements, while h3k14ac marks a subset of inactive inducible promoters in mouse embryonic stem cells. BMC genomics **13**(1), 1–18 (2012)

[20] Calo, E., Wysocka, J.: Modification of enhancer chromatin: what, how, and why? Molecular cell **49**(5), 825–837 (2013)

[21] Zhang, T., Zhang, Z., Dong, Q., Xiong, J., Zhu, B.: Histone h3k27 acetylation is dispensable for enhancer activity in mouse embryonic stem cells. Genome biology **21**(1), 1–7 (2020)

[22] Song, L., Crawford, G.E.: Dnase-seq: a high-resolution technique for mapping active gene regulatory elements across the genome from mammalian cells. Cold Spring Harbor Protocols **2010**(2), 5384 (2010)

[23] Barski, A., Cuddapah, S., Cui, K., Roh, T.-Y., Schones, D.E., Wang, Z., Wei, G., Chepelev, I., Zhao, K.: High-resolution profiling of histone methylations in the human genome. Cell **129**(4), 823–837 (2007)

[24] Ferrari, K.J., Scelfo, A., Jammula, S., Cuomo, A., Barozzi, I., Stützer, A.,

Fischle, W., Bonaldi, T., Pasini, D.: Polycomb-dependent h3k27me1 and h3k27me2 regulate active transcription and enhancer fidelity. Molecular cell **53**(1), 49–62 (2014)

[25] Schwartz, S., Meshorer, E., Ast, G.: Chromatin organization marks exon-intron structure. Nature structural & molecular biology **16**(9), 990 (2009)

[26] Li, T., Liu, Q., Garza, N., Kornblau, S., Jin, V.X.: Integrative analysis reveals functional and regulatory roles of h3k79me2 in mediating alternative splicing. Genome medicine **10**(1), 1–11 (2018)

[27] Farooq, Z., Banday, S., Pandita, T.K., Altaf, M.: The many faces of histone h3k79 methylation. Mutation Research/Reviews in Mutation Research **768**, 46–52 (2016)

[28] Vig, J., Madani, A., Varshney, L.R., Xiong, C., richard socher, Rajani, N.: {BERT}ology meets biology: Interpreting attention in protein language models. In: International Conference on Learning Representations (2021). https://openreview.net/forum?id=YWtLZvLmud7

[29] Raganato, A., Tiedemann, J., *et al.*: An analysis of encoder representations in transformer-based machine translation. In: Proceedings of the 2018 EMNLP Workshop BlackboxNLP: Analyzing and Interpreting Neural Networks for NLP (2018). The Association for Computational Linguistics

[30] Kovaleva, O., Romanov, A., Rogers, A., Rumshisky, A.: Revealing the dark secrets of bert. arXiv preprint arXiv:1908.08593 (2019)

[31] Lee, J., Shin, J.-H., Kim, J.-S.: Interactive visualization and manipulation of attention-based neural machine translation. In: Proceedings of the 2017 Conference on Empirical Methods in Natural Language Processing: System Demonstrations, pp. 121–126 (2017)

[32] Serrano, S., Smith, N.A.: Is attention interpretable? In: Proceedings of the 57th Annual Meeting of the Association for Computational Linguistics, pp. 2931–2951 (2019)

[33] Jain, S., Wallace, B.C.: Attention is not explanation. arXiv preprint arXiv:1902.10186 (2019)

[34] Howe, K.L., Achuthan, P., Allen, J., Allen, J., Alvarez-Jarreta, J., Amode, M.R., Armean, I.M., Azov, A.G., Bennett, R., Bhai, J., Billis, K., Boddu, S., Charkhchi, M., Cummins, C., Da Rin Fioretto, L., Davidson, C., Dodiya, K., El Houdaigui, B., Fatima, R., Gall, A., Garcia Giron, C., Grego, T., Guijarro-Clarke, C., Haggerty, L., Hemrom, A., Hourlier, T., Izuogu, O.G., Juettemann, T., Kaikala, V., Kay, M.,

Lavidas, I., Le, T., Lemos, D., Gonzalez Martinez, J., Marugán, J.C., Maurel, T., McMahon, A.C., Mohanan, S., Moore, B., Muffato, M., Oheh, D.N., Paraschas, D., Parker, A., Parton, A., Prosovetskaia, I., Sakthivel, M.P., Salam, A., Schmitt, B.M., Schuilenburg, H., Sheppard, D., Steed, E., Szpak, M., Szuba, M., Taylor, K., Thormann, A., Threadgold, G., Walts, B., Winterbottom, A., Chakiachvili, M., Chaubal, A., De Silva, N., Flint, B., Frankish, A., Hunt, S.E., IIsley, G.R., Langridge, N., Loveland, J.E., Martin, F.J., Mudge, J.M., Morales, J., Perry, E., Ruffier, M., Tate, J., Thybert, D., Trevanion, S.J., Cunningham, F., Yates, A.D., Zerbino, D.R., Flicek, P.: Ensembl 2021. Nucleic Acids Research **49**(D1), 884–891 (2020) https://academic.oup.com/nar/article-pdf/49/D1/D884/35364073/gkaa942.pdf. https://doi.org/10.1093/nar/gkaa942

[35] Shilatifard, A.: Chromatin modifications by methylation and ubiquitination: implications in the regulation of gene expression. Annu. Rev. Biochem. **75**, 243–269 (2006)

[36] Boyle, A.P., Davis, S., Shulha, H.P., Meltzer, P., Margulies, E.H., Weng, Z., Furey, T.S., Crawford, G.E.: High-resolution mapping and characterization of open chromatin across the genome. Cell **132**(2), 311–322 (2008)

[37] Robertson, A.G., Bilenky, M., Tam, A., Zhao, Y., Zeng, T., Thiessen, N., Cezard, T., Fejes, A.P., Wederell, E.D., Cullum, R., *et al.*: Genome-wide relationship between histone h3 lysine 4 mono-and tri-methylation and transcription factor binding. Genome research **18**(12), 1906–1917 (2008)

[38] Mohn, F., Schübeler, D.: Genetics and epigenetics: stability and plasticity during cellular differentiation. Trends in Genetics **25**(3), 129–136 (2009)

[39] Voita, E., Talbot, D., Moiseev, F., Sennrich, R., Titov, I.: Analyzing multi-head self-attention: Specialized heads do the heavy lifting, the rest can be pruned. In: Proceedings of the 57th Annual Meeting of the Association for Computational Linguistics, pp. 5797–5808 (2019)

[40] Schreiber, J., Boix, C., Li, H., Guan, Y., Chang, C.-C., Chang, J.-C., Hawkins-Hooker, A., Schölkopf, B., Schweikert, G., Carulla, M.R., *et al.*: The encode imputation challenge: a critical assessment of methods for cross-cell type imputation of epigenomic profiles. Genome Biology **24**(1), 1–22 (2023)
